# Understanding the group dynamics and success of teams

**DOI:** 10.1098/rsos.160007

**Published:** 2016-04-06

**Authors:** Michael Klug, James P. Bagrow

**Affiliations:** 1 Department of Mathematics and Statistics, The University of Vermont, Burlington, VT, USA; 2 Vermont Complex Systems Center, The University of Vermont, Burlington, VT, USA; 3 Vermont Advanced Computing Core, The University of Vermont, Burlington, VT, USA

**Keywords:** teamwork, collective dynamics, data science, open source software

## Abstract

Complex problems often require coordinated group effort and can consume significant resources, yet our understanding of how teams form and succeed has been limited by a lack of large-scale, quantitative data. We analyse activity traces and success levels for approximately 150 000 self-organized, online team projects. While larger teams tend to be more successful, workload is highly focused across the team, with only a few members performing most work. We find that highly successful teams are significantly more focused than average teams of the same size, that their members have worked on more diverse sets of projects, and the members of highly successful teams are more likely to be core members or ‘leads’ of other teams. The relations between team success and size, focus and especially team experience cannot be explained by confounding factors such as team age, external contributions from non-team members, nor by group mechanisms such as social loafing. Taken together, these features point to organizational principles that may maximize the success of collaborative endeavours.

## 1. Introduction

Massive datasets describing the activity patterns of large human populations now provide researchers with rich opportunities to quantitatively study human dynamics [1,2], including the activities of groups or teams [3,4]. New tools, including electronic sensor systems, can quantify team activity and performance [5,4]. With the rise in prominence of network science [6,7], much effort has gone into discovering meaningful groups within social networks [8–15] and quantifying their evolution [15,16]. Teams are increasingly important in research and industrial efforts [3,4,17–21], and small, coordinated groups are a significant component of modern human conflict [22,23]. There are many important dimensions along which teams should be studied, including their size, how work is distributed among their members, and the differences and similarities in the experiences and backgrounds of those team members. Recently, there has been much debate on the ‘group size hypothesis’ that larger groups are more robust or perform better than smaller ones [24–27]. Scholars of science have noted for decades that collaborative research teams have been growing in size and importance [20,28–30]. At the same time, however, social loafing, where individuals apply less effort to a task when they are in a group than when they are alone, may counterbalance the effectiveness of larger teams [31–33]. Meanwhile, case studies show that leadership [3,34–36] and experience [37,38] are key components of successful team outcomes, while specialization and multitasking are important but potentially error-prone mechanisms for dealing with complexity and cognitive overload [39,40]. In all of these areas, large-scale, quantitative data can push the study of teams forward.

Teams are important for modern software engineering tasks, and researchers have long studied the digital traces of open source software projects to better quantify and understand how teams work on software projects [41,42]. Researchers have investigated estimators of work activity or effort based on edit volume, such as different ways to count the number of changes made to a software's source code [43–46]. Various dimensions of success of software projects such as popularity, timeliness of bug fixes or other quality measures have been studied [47–49]. Successful open source software projects show a layered structure of primary or core contributors surrounded by lesser, secondary contributors [50]. At the same time, much work is focused on case studies [45,51] of small numbers of highly successful, large projects [41]. Considering these studies alone runs the risk of survivorship bias or other selection biases, so large-scale studies of large quantities of teams are important complements to these works.

Users of the GitHub web platform can form teams to work on real-world projects, primarily software development but also music, literature, design work and more. A number of important scientific computing resources are now developed through GitHub, including astronomical software, genetic sequencing tools and key components of the Compact Muon Solenoid experiment's data pipeline.^1^ A ‘GitHub for science’ initiative has been launched^2^ and GitHub is becoming the dominant service for open scientific development.

GitHub provides rich public data on team activities, including when new teams form, when members join existing teams and when a team's project is updated. GitHub also provides social media tools for the discovery of interesting projects. Users who see the work of a team can choose to flag it as interesting to them by ‘starring’ it. The number of these ‘stargazers’ *S* allows us to quantify one aspect of the *success* of the team, in a manner analogous to the use of citations of research literature as a proxy for ‘impact’ [52]. Of course, as with bibliometric impact, one should be cautious and not consider success to be a perfectly accurate measure of *quality*, something that is far more difficult to objectively quantify. Instead this is a measure of popularity as would be other statistics such as web traffic, number of downloads and so forth [47].

In this study, we analyse the memberships and activities of approximately 150 000 teams, as they perform real-world tasks, to uncover the blend of features that relate to success. To the best of our knowledge this is the largest study of real-world team success to date. We present results that demonstrate (i) how teams distribute or focus work activity across their members, (ii) the mixture of experiential diversity and collective leadership roles in teams, and (iii) how successful teams are different from other teams while accounting for confounds such as team size.

The rest of this paper is organized as follows: in §2, we describe our GitHub dataset; give definitions of a team, team success and work activity/focus of a team member; and introduce metrics to measure various aspects of the experience and experiential diversity of a team's members. In §3, we present our results relating these measures to team success. In §4, we present statistical tests on linear regression models of team features to control for potential confounds between team features and team success. Lastly, we conclude with a discussion in §5.

## 2. Material and methods

### 2.1 Dataset and team selection

Public GitHub data covering 1 January 2013 to 1 April 2014 was collected from githubarchive.org in April 2014. In their own words, ‘GitHub Archive is a project to record the public GitHub timeline, archive it, and make it easily accessible for further analysis’. These activity traces contain approximately 110M unique events, including when users create, join, or update projects. Projects on GitHub are called ‘repositories’. For this work, we define a *team* as the set of users who can directly update (push to) a repository. These users constitute the *primary* team members as they have either created the project or been granted autonomy to work on the project. The number of team members was denoted by *M*. Activity or workload *W* was estimated from the number of pushes. A push is a bundle of code updates (known as commits), however most pushes contain only a single commit (electronic supplementary material; see also [46]). As with all studies measuring worker effort from lines-of-code metrics, this is an imperfect measure as the complexity of a unit of work does not generally map to the quantity of edits. Users on GitHub can bookmark projects they find interesting. This is called ‘stargazing’. We take the maximum number of stargazers for a team as its measure of *success* *S*. This is a popularity measure of success; however, the choice to bookmark a project does imply it offers some value to the user. To avoid abandoned projects, studied teams have at least one stargazer (*S*>0) and at least two updates per month on average within the githubarchive data. These selection criteria leave *N*=151 542 teams. We also collect the time of creation on GitHub for each team project. This is useful for measuring confounds: for example, older teams may tend to have both more members and more opportunities to increase success. Of the teams studied, 67.8% were formed within our data window. Beyond considering team age as a potential confounder, we do not study temporal dynamics such as team formation in this work. A small number of studied teams (1.08%) have more than 10 primary members (*M*>10); those teams were not shown in figures, but they were present in all statistical analyses. Lastly, to ensure our results are not due to outliers, in some analyses we excluded teams above the 99th percentile of *S*. Despite a strong skew in the distribution of *S*, these highly popular teams account for only 2.54% of the total work activity of the teams considered in this study (2.27% when considering teams with *M*≤10 members).

#### 2.1.1 Secondary team

GitHub provides a mechanism for external, non-team contributors to propose work that team members can then choose to use or not. These proposals are called pull requests. (Other mechanisms, such as discussions about issues, are also available to non-team contributors.) These secondary or external team contributors are not the focus of this work and have already been well studied by OSS researchers [41]. However, it is important to ensure that they do not act as confounding factors for our results, as more successful teams will tend to have more secondary contributions than other teams. So we measure for each team *M*_ext_, the number of unique users who submit at least one pull request, and *W*_ext_, the number of pull requests. We will include these measures in our combined regression models. Despite their visibility in GitHub, pull requests are rare [53]; in our data, 57.7% of teams we study have *W*_ext_=0, and when present pull requests are greatly outnumbered by pushes on average: 〈*W*/*W*_ext_|*W*_ext_>0〉=42.3 (median 16.0), averaged over all teams with at least one pull request.

### 2.2 Effective team size

The number of team members, *M*, does not fully represent the size of a team as the distribution of work may be highly skewed across team members. To capture the *effective team size* *m*, accounting for the relative contribution levels of members, we use *m*=2^*H*^, where $H=-\sum_{i=1}^{M}f_{i}\log_{2}f_{i}$, and *f*_*i*_=*w*_*i*_/*W* is the fraction of work performed by team member *i*. This gives *m*=*M* when all *f*_*i*_=1/*M*, as expected. This simple, entropic measure is known as perplexity in linguistics and is closely related to species diversity indices used in ecology and the Herfindahl–Hirschman index used in economics.

### 2.3 Experience, diversity and leads

Denote with *R*_*i*_ the set of projects that user *i* works on (has pushed to). (Projects in *R*_*i*_ need at least twice-monthly updates on average, as before, but may have *S*=0 so as to better capture *i*'s background, not just successful projects.) We estimate the *experience* *E* of a team of size *M* as

$$E=\frac{1}{M}\sum_{i}|R_{i}|-1$$

and the experiential *diversity* *D* as

$$D=\frac{|\underset{i}{⋃}R_{i}|}{\sum_{i}|R_{i}|},$$

where the sums and union run over the *M* members of the team. Note that *D*∈[1/*M*,1). Experience measures the quantity of projects the team works on while diversity measures how many or how few projects the team members have in common, the goal being to capture how often the team has worked together. Lastly, someone is a *lead* when, for at least one project they work on, they contribute more work to that project than any other member. A non-lead member of team *j* may be the lead of project *k*≠*j*. The number of leads *L*_*k*_ in team *k* of size *M*_*k*_ is

$$L_{k}=\sum_{i=1}^{M_{k}}\min\left(\sum_{j}L_{ij},1\right),$$

where *L*_*ij*_=1 if user *i* is the lead of team *j*, and zero otherwise. The first sum runs over the *M*_*k*_ members of team *k*, the second runs over all projects *j*. Of course, the larger the team the more potential leads it may contain so when studying the effects of leads on team success we only compare teams of the same size (comparing *L* while holding *M* fixed). Otherwise, *E* and *D* already account for team size.

## 3. Results

**Figure 1.**
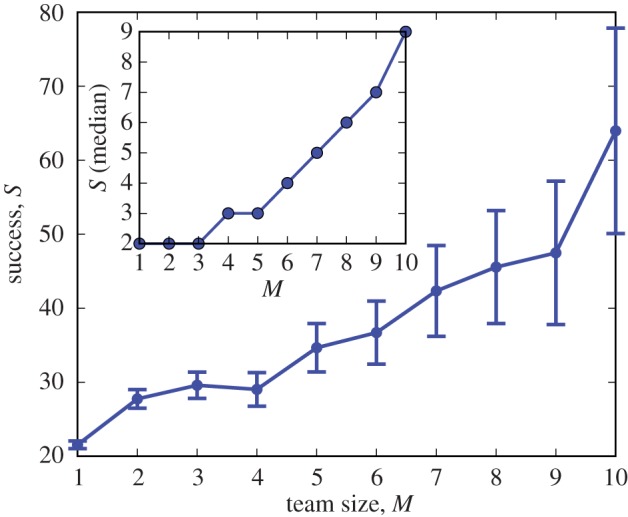
Larger teams have significantly more success on average, with a 300% increase in *S* as *M* goes from 1 to 10. This correlation may be due to more team members driving project success or success may act as a mechanism to recruit team members. Error bars here and throughout denote ±1.96 s.e. (Inset) Using the median instead of the mean shows that this trend is not due to outliers.

We began our analysis by measuring team success *S* as a function of team size *M*, the number of primary contributors to the team's project. As *S* is, at least partially, a popularity measure, we expect larger teams to also be more successful. Indeed, there was a positive and significant relationship (*p*<10^−10^, rank correlation *ρ*=0.0845) between the size of a team and its success, with 300% greater success on average for teams of size *M*=10 compared with solos with *M*=1 (figure 1). This strong trend also holds for the median success (inset). While this observed trend was highly significant, the rank correlation *ρ* indicates that there remains considerable variation in *S* that is not captured by team size alone.

Our next analysis reveals an important relationship between team focus and success. Unlike bibliographic studies, where teams can only be quantified as the listed coauthors of a paper, the data here allow us to measure the intrinsic work or volume of contributions from each team member to the project. For each team we measured the contribution *w*_*r*_ of a member to the team's ongoing project, how many times that member updated the project (see Material and methods). Team members were ranked by contribution, so *w*_1_ counts the work of the member who contributed the most, *w*_2_ the second heaviest contributor and so forth. The total work of a team is $W=\sum_{r=1}^{M}w_{r}$.

**Figure 2.**
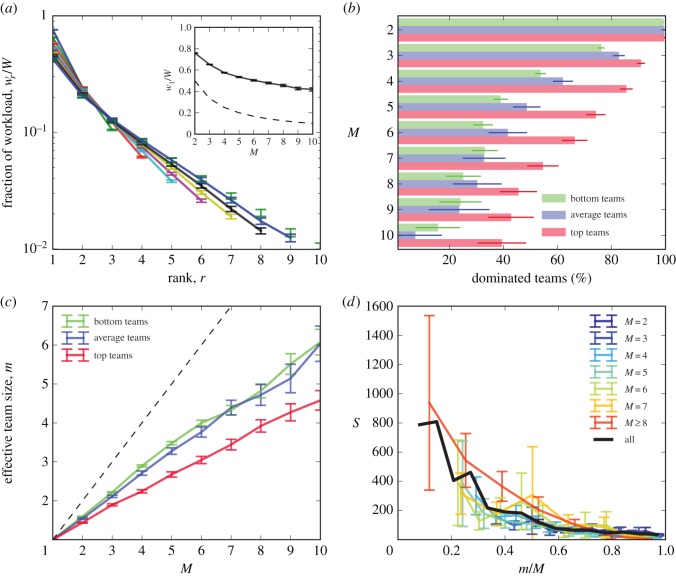
Teams are focused, and top teams are more focused than other teams of the same size. (*a*) The average fraction of work *w*_*r*_/*W* performed by the *r*th most active member, where *W* is the total work of the team, for different size teams. Larger teams perform more work overall, but the majority of work is always done by a small subset of the *M* members (note the logarithmic axis). Inset: the fraction of work performed by the most active team member is always high, often larger than half the total. The dashed line indicates the lower bound of uniform work distribution, *w*_*r*_/*W*=1/*M*. (*b*) A team is *dominated* when the most active member does more work than all other members combined. Top teams are significantly more probably to be dominated than either average teams or bottom teams for all *M*>2. (*Top team*: above the 90th percentile in *S*; *average team*: greater than the 40th percentile of *S* and less than or equal to the 60th percentile of *S*; *bottom team*: at or below the 10th percentile of *S*.) (*c*) The effective team size *m* (see Material and methods), a measure that accounts for the skewed distribution of work in (*a*), is significantly smaller than *M*. Moreover, top teams are significantly more focused, having smaller effective sizes, than average or bottom teams at all sizes *M*>1. This includes the case *M*=2, which did not show a significant difference in (*b*). The dashed line denotes the upper bound *m*=*M*. (*d*) Success is universally higher for teams with smaller *m*/*M*, independent of *M*, further supporting the importance of focused workloads. The solid lines indicates the average trend for all teams 2≤*M*≤10. These results are not due to outliers in *S*; see the electronic supplementary material.

We found that the distribution of work over team members showed significant skew, with *w*_1_ often more than two to three times greater than *w*_2_ (figure 2*a*; electronic supplementary material). This means that the workloads of projects are predominantly carried by a handful of team members, or even just a single person. Larger teams perform more total work, and the heaviest contributor carries much of that effort: the inset of figure 2*a* shows that *w*_1_/*W*, the fraction of work carried by the rank one member, falls slowly with team size, and is typically far removed from the lower bound of equal work among all team members. See the electronic supplementary material for more details. This result is in line with prior studies [51], supporting the plausibility of our definition of a team and our use of pushes to measure work.

This focus in work activity indicates that the majority of the team serves as a support system for a core set of members. Does this arrangement play a role in whether or not teams are successful? We investigated this in several ways. First, we asked whether or not a team was *dominated*, meaning that the lead member contributed more work than all other members combined ($w_{1}/W>\frac{1}{2}$). Highly successful ‘top’ teams, those in the top 10% of the success distribution, were significantly more likely to be dominated than average teams, those in the middle 20% of *S*, or ‘bottom’ teams, those in the bottom 10% of the *S* (figure 2*b*).

Can this result be due to a confounding effect from success? More successful projects will tend to have more external contributors, for example, which can change the distribution of work. For example, in one scenario a team member may be a ‘community manager’ merging in large numbers of external contributions from non-team members. To test this we examined only the 57.7% of teams that had no external contributions (*W*_ext_=0) and tested among only those teams whether dominated teams were more successful than non-dominated teams. Within this subset of teams, dominated teams had significantly higher *S* than non-dominated teams (Mann–Whitney *U* test (MWU) with continuity correction, *p*<10^−8^). The MWU is non-parametric, using ranks of (in this case) *S* to mitigate the effects of skewed data, and does not assume normality. We conclude from this that external contributions do not fully explain the relationship between workload focus and team success.

Next, we moved beyond the effects of the heaviest contributor by performing the following analysis. For each team we computed its *effective* team size *m*, directly accounting for the skew in workload (see Material and methods for full details). This effective size can be roughly thought of as the average number of unique contributors per unit time and need not be a whole number. For example, a team of size *M*=2 where both members contribute equally will have effective size *m*=2, but if one member is responsible for 95% of the work the team would have *m*≈1.22. Note that *M* and *m* are positively correlated (*ρ*=0.985).

Figure 2*c* shows that (i) all teams are effectively much smaller than their total size would indicate, for all sizes *M*>1, and (ii) top teams are significantly smaller in effective size (and therefore more focused in their work distribution) than average or bottom teams with the same *M*. Further, success is significantly, negatively correlated with *m*, for all *M* (figure 2*d*). More focused teams have significantly more success than less focused teams of the same size, regardless of total team size.

Further analyses revealed the importance of team composition and its role in team success.

Team members do not perform their work in a vacuum, they each bring experiences from their other work. Often members of a team will work on other projects. We investigated these facets of a team's composition by exploring (i) how many projects the team's members have worked on, (ii) how diverse the other projects are (whether the team members have many or few other projects in common) and (iii) how many team members were ‘leads’ of other projects.

An estimate of experience, *E*, the average number of other projects that team members have worked on (see Material and methods), was significantly related to success. However, the trend was not particularly strong (see the electronic supplementary material) and, as we later show via combined modelling efforts, this relationship with success was entirely explainable by the teams' other measurable quantities.

It may be that the volume of experience does not contribute much to the success of a team, but this seems to contradict previous studies on the importance of experience and wisdom [37,38]. To investigate, we turned to a different facet of a team's composition, the diversity of the team's background. Successful teams may tend to be composed of members who have frequently worked together on the same projects in the past, perhaps developing an experiential shorthand. Conversely, successful teams may instead have multiple distinct viewpoints, solving challenges with a multi-disciplinary perspective [54].

**Figure 3.**
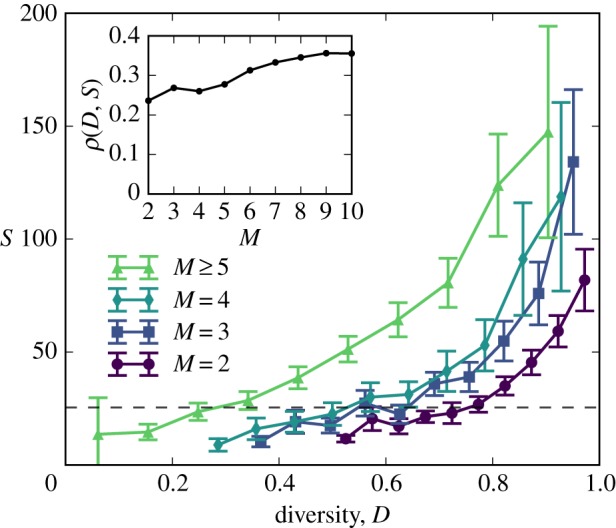
Teams whose members belong to more diverse sets of other teams tend to be more successful, regardless of team size. The dashed line denotes the average success of all teams. (Inset) The rank correlation *ρ* between diversity and success grows with team size. Teams above the 99th percentile in *S* were excluded to ensure the trend is not due to outliers.

To estimate the distinctness of team member backgrounds, the diversity *D* was measured as the fraction of projects that team members have worked on that are unique (see Material and methods). Diversity is low when all *M* members have worked on the same projects together (*D*=1/*M*), but *D* grows closer to 1 as their backgrounds become increasingly diverse. A high team diversity was significantly correlated with success, regardless of team size (figure 3). Even small teams seem to have benefited greatly from diversity: high-*D* duos averaged nearly *eight times* the success of low-*D* duos. The relationship between *D* and *S* was even stronger for larger teams (figure 3, inset), implying that larger teams can more effectively translate this diversity into success. Even if the raw volume of experience a team has does not play a significant role in the team's success, the diversity of that experience was significantly correlated with team success. See also our combined modelling efforts.

Considerable attention has been paid recently to collective leadership, where decision-making structures emerge from the mass of the group instead of being imposed via a top-down hierarchy [34,36]. The open collaborations studied here have the potential to display collective leadership due to their volunteer-driven, self-organized nature. The heaviest contributor to a team is most likely to occupy such a leadership role. Further, as teams overlap, a secondary member of one team may be the ‘lead,’ or heaviest contributor to another. This poses an interesting question: Even though teams are heavily focused, are teams more successful when they contain many leads, or few? A team with many leads will bring considerable experience, but most of its members may also be unable to dedicate their full attention to the team.

**Figure 4.**
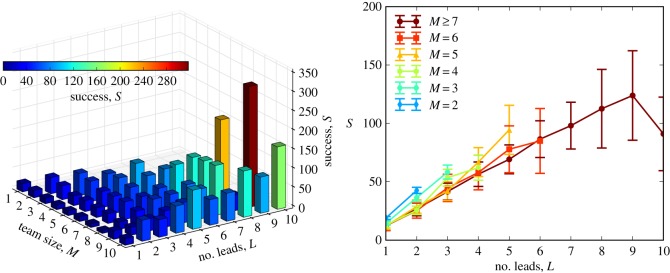
Teams with more leads have higher success than teams of the same size with fewer leads. A lead is someone who contributes more work to at least one team he or she belongs to than any other members of that team. Outliers in *S* were removed as before.

To answer this, we measured *L*, the number of team members who are the lead of at least one project (1≤*L*≤*M*, see Material and methods) and found that teams with many leads have significantly higher success than teams *of the same size* with fewer leads (figure 4). Only one team member can be the primary contributor to the team, so a team can only have many leads if the other members have focused their work activity on other projects. Team members who are focused on other projects can potentially only provide limited support, yet successful teams tend to arrange their members in exactly this fashion. Of course, the strong focus in work activity (figure 2) is probably interrelated with these observations. However, we will soon show that both remain significantly related to success in combined models.

Expanding on this observation, table 1 illustrates the extreme case of teams of size *M* with a single lead (*L*=1) compared with teams of the same size composed entirely of leads (*L*=*M*). The latter always displayed significantly higher success than the former (MWU test, see table 1), independent of team size, underscoring the correlations displayed in figure 4. Often the difference was massive: teams of size *M*=7, for example, averaged more than 1200% higher success when *L*=7 than when *L*=1.

**Table 1.** Teams composed entirely of leads (*L*=*M*) are significantly more successful (MWU test on *S*) than teams of the same size with one lead (*L*=1), regardless of team size *M*. Teams above the 99th percentile in *S* were excluded to ensure the differences were not due to outliers.

	no. teams *N*		mean success *S*		
*M*	*L*=1	*L*=*M*	*L*=1	*L*=*M*	MWU *p*-value
2	14 823	8894	18.9	42.5	<10^−213^
3	6171	2261	14.5	58.3	<10^−210^
4	3063	717	12.8	62.1	<10^−112^
5	1489	289	12.1	94.5	<10^−55^
6	740	124	12.3	85.0	<10^−36^
7	350	46	9.8	120.5	<10^−15^
8	179	19^*a*^	7.5	224.1	<10^−8^
9	125	9	22.2	316.8	<0.008
10	66	6	17.8	163.5	<0.005

^*a*^When *M*≥8, the number of teams with *L*=*M* is too small (*N*<20) for us to reasonably conclude the difference in *S* is significant, despite the small *p*-values.

These results on team composition cannot be easily explained as a confound with success or secondary contributions as they study specific features and projects of the individuals who comprise a team, those features are not related to the successes of other projects an individual may work on, and they strictly control for total team size *M* (e.g. we only compare teams with different values of *L* when they have the same value *M*). These results further amplify our findings on team focus, and augment important existing research [3,4,36,37,54].

Taken together, our results demonstrate that successful teams tend to be focused (figure 2), successful teams tend to be experientially diverse (figure 3) and successful teams tend to have many leads (figure 4). We have found that teams tend to do best when optimized along all three of these dimensions. Of course, it is necessary to explore the joint effects of quantities, to see if one relationship can be explained by another, which we will do with multivariate statistical models.

## 4. Combined models and confounds

One important aspect of the individual team measurements is that they do not exist in isolation. For example, successful teams also have high work activity (high *W*). This can correlate with effective team size *m* as the potential inequality between team members can grow as their total activity grows. In other words, we need to see how our team measures relate to success *together*.

To understand the relative effects of these team composition measures, we fitted a linear regression model of success as a function of all explored measures (table 2). Not only did this regression allow us to determine whether a variable was significant or if it was confounded by the other measures, but the coefficients (on the standardized variables) let us measure the relative strengths of each variable. We also included the age of a project *T* (measured as the time difference between the recorded creation time of the project and the end of our data window; see Material and methods) as this may also be a potential confounding factor (older projects have had more time to gain members and to gain success).

**Table 2.** OLS regression model on team success, *S*=*α*+*β*_*M*_*M*+*β*_*m*_*m*+*β*_*W*_*W*+*β*_*E*_*E*+ *β*_*D*_*D*+*β*_*L*_*L*+*β*_*T*_*T*. Outliers (above the 99th percentile in *S*) were filtered out to ensure they do not skew the model.

variable *x*	coefficient $\beta_{x}^{a}$	*p*-value
constant, *α*	1.351×10^−14^±0.004951	1
team size, *M*	0.0848±0.013963	<10^−31^
eff. team size, *m*	−0.0989±0.012140	<10^−56^
total work, *W*	0.0323±0.004997	<10^−35^
experience, *E*	0.0004068±0.004985	0.8729
diversity, *D*	0.04099±0.006357	<10^−35^
no. leads, *L*	0.1388±0.006921	0
age, *T*	0.1273±0.005014	0

^*a*^Variables are standardized for comparison such that a coefficient *β*_*x*_ implies that increasing a variable *x* by one standard deviation *σ*_*x*_ corresponds to a *β*_*x*_*σ*_*S*_ increase in *S*, holding other variables fixed.

Examining the regression coefficients showed that the number of leads *L* was the variable most strongly correlated with team success. Team age *T*, effective team size *m* and team size *M* play the strongest roles after *L* in team success, and all three were also significant in the presence of the other variables. The coefficient on *m* was negative while for *M* it was positive, further underscoring our result that, while teams should be big, they effectively should be small. Next, the diversity *D* of the team, followed by the total work *W* done on the project, were also significant measures related to success. Finally, overall team experience *E* was not significant in this model (*p*>0.1). We conclude that, while *S* and *E* are correlated by themselves, any effects of *E* are explained by the other quantities.

What about secondary contributions, those activities made by individuals outside the primary team? We already performed one test showing that dominated teams are more successful than non-dominated teams even when there are no secondary contributions. Continuing along these lines, we augmented this linear model with two more dependent variables, *M*_ext_ and *W*_ext_. Regressing on this expanded model (see the electronic supplementary material for details) did not change the significance of any coefficients at the *p*=0.05 level; *E* remained insignificant (*p*>0.1). Both new variables were significant (*p*<0.05). Note that there were no multicollinearity effects in either regression model (condition numbers less than 10). We conclude that secondary contributions cannot alone explain the observations relating team focus, experience and lead number to team success.

## 5. Discussion

There has been considerable debate concerning the benefits of specialization compared with diversity in the workplace and other sectors [39]. Our discoveries here show that a high-success team forms a diverse support system for a specialist core, indicating that both specialization and diversity contribute to innovation and success. Team members should be both specialists, acting as the lead contributor to a team, and generalists, offering ancillary support for teams led by another member. This has implications when organizations are designing teams and wish to maximize their success, at least as success was measured in these data. Teams tend to do best on average when they maximize *M* (figure 1*b*) while minimizing *m* (figure 2*d*) and maximizing *D* (figure 3) and *L* (figure 4).

Of course, some tasks are too large for a single person or small team to handle, necessitating the need for mega teams of hundreds or even thousands of members. Our results imply that such teams may be most effective when broken down into large numbers of small, overlapping groups, where all individuals belong to a few teams and are the lead of at least one. Doing so will help maximize the experiential diversity of each sub-team, while ensuring each team has someone ‘in charge’. An important open question is what the best ways are to design such pervasively overlapping groups [14], a task that may be project- or domain-specific but which is worth further exploration.

The negative relationship between effective team size *m* and success *S* (as well as the significantly higher presence of dominated teams among high success teams) further belies the myth of multitasking [39] and supports the ‘surgical team’ arguments of Brooks [17]. Focused work activity, often by even a single person, is a hallmark of successful teams. This focus both limits the cognitive costs of task switching, and lowers communication and coordination barriers, as so much work is being accomplished by one or only a few individuals. We have provided statistical tests demonstrating that the relationship between focus and success cannot be due to secondary/external team contributions alone.

Work focus could possibly be explained by *social loafing* where individual members of a group contribute less effort as part of the group than they would alone, yet loafing does not explain the correlation between e.g. leads and success (figure 4). Likewise, our team composition results on group experience, experiential diversity and the number of leads cannot be easily explained as a confound with success or secondary contributions: they study specific features of the individuals who comprise a team, those features are not related to the successes of other projects an individual may work on, and they strictly control for total team size *M* (except for the number of leads *L*, so for that measure we only compared teams with the same *M*). The measures we used for external team contributions, *M*_ext_ and *W*_ext_, may be considered measures of success themselves, and studying or even predicting their levels from team features may prove a fruitful avenue of future work.

Lastly, there are two remaining caveats worth mentioning. We do not specifically control for automatically mirrored repositories (where a computer script copies updates to GitHub). Accurately detecting such projects at scale is a challenge beyond the scope of this work. However, we expect most will either be filtered out by our existing selection criteria or else they will probably only have a single (automated) user that only does the copying. The second concern is work done outside of GitHub or, more generally, mismatched assignments between usernames and their work. This is also challenging to fully address (one issue is that the underlying git repository system does not authenticate users). We acknowledge this concern for our workload focus results, but even it cannot explain the significant trends we observed on team composition such as the density of leads. Noise due to improperly recorded or ‘out-of-band’ work has in principle affected all quantitative studies of online software repositories.

## Supplementary Material

Supplementary Information

## Footnotes

1 For examples, see https://github.com/showcases/science.

2 See https://github.com/blog/1840-improving-github-for-science.

## Data accessibility

All data analysed are made publicly available by the GitHub Archive Project (https://www.githubarchive.org).
