# Supplementary material for: Understanding the group dynamics and success of teams
Source: R Soc Open Sci. 2016 Apr 6;3(4):160007. doi: 10.1098/rsos.160007 (PMC4852640; doi:10.1098/rsos.160007)
Supplement: Supplementary Information [file rsos160007supp1.pdf]

Supporting Information

Understanding the group dynamics and success of teams
by Michael Klug and James P. Bagrow

Table of Contents

S1 GitHub popularity 1

S2 Number of commits per push 1

S3 The distribution of workload across team members 1

S4 Outliers in success do not skew team focus results 2

S5 Experience is weakly related to team success 3

S6 Linear model with secondary team contributions 3

List of Figures

S1 GitHub is becoming the dominant platform for open development and collaboration 2
S2 Most GitHub pushes consist of only one commit. 2
S3 Larger teams are more active but all teams distribute work the same way 3
S4 Teams focus trends are not due to outliers in S 4
S5 Experience weakly correlates with success 5

S1 GitHub popularity

Since roughly 2011 GitHub has become the predominant online platform for developing and hosting open source software and other projects. Competitors such as Sourceforge have been left behind. Figure S1 shows the Google search volume for GitHub and its competitors, as gathered from Google Trends (`https://www.google.com/trends/`).

S2 Number of commits per push

A "push" to GitHub can consist of multiple "commits". A commit is an individual update to the project's git repository. However, most pushes consist of only a single commit (Fig. S2). A commit can contain many or few changes to a repository, and a change eventually affecting only a small piece of a file may have taken an enormous amount of effort to achieve. This variability makes "lines of code" work metrics noisy. Despite this, they are a commonly analyzed metric.

S3 The distribution of workload across team members

Figure S3A shows how the total workload (number of pushes) of teams grows with team size M, meaning larger teams are more active in total. This makes sense. The inset shows how the fraction of total work done

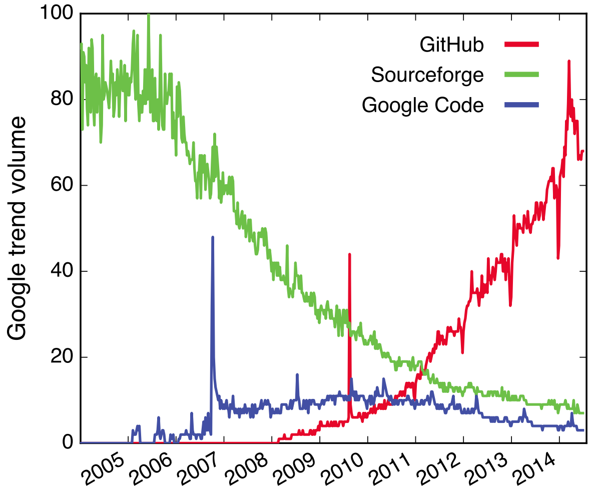

Figure S1: GitHub is becoming the dominant platform for open development and collaboration. Google trends (normalized) search volume over time for searches "GitHub", "Sourceforge", and "Google Code". This trend data serves as a proxy for popularity.

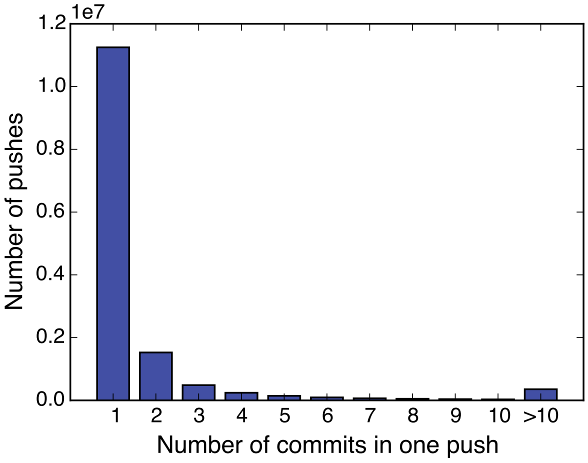

Figure S2: Most GitHub pushes consist of only one commit.

by the lead (w_1/W) decreases on average as team size grows. The lower bound $1/M$ is almost never reached, indicating that workloads remain "front-loaded" for all team sizes.

Figure S3B shows how the average workload per team member *decreases* as teams grow. The ten curves correspond to deciles of the total workload distribution. They all follow the same functional form, except possibly the highest decile which decays slightly more slowly. This indicates that the distribution of workload across team members is independent of total workload; more active teams distribute their workloads across their members in the same manner as less active teams.

S4 Outliers in success do not skew team focus results

In Fig. S4 we reproduce the results corresponding to main text Fig. 2 but withhold the 1% highest S projects. Our results do not change, indicating that the trends observed in main text Fig. 2 are not due to outliers. Note that the magnitude of the trend in figure panel D does decrease quite a bit, but the trend is still evident.

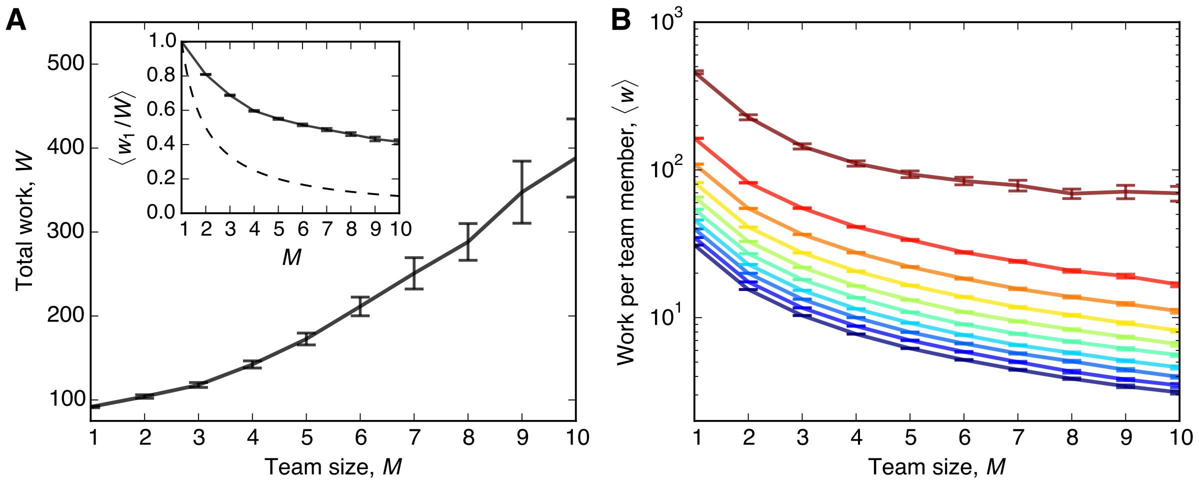

Figure S3: Larger teams are more active but all teams distribute work the same way. (**A**) The total work (total number of pushes) grows with team size, on average. (Inset) The average fraction of work done by the lead decreases with team size, but on average remains well above the lower bound $1/M$. (**B**) The average work per team member decreases as team size grows. The ten curves correspond to the deciles of the total work distribution. The functional form is identical for each decile, except perhaps the highest decile which decays slightly more slowly with M, indicating that the way in which work is distributed over a team is independent of the total activity of that team.

S5 Experience is weakly related to team success

One of the quantities we used to understand a team is their experience E, defined as the average number of other teams that members of the team belong to. We found in the main text that E and S are significantly correlated by themselves, but that E is not significant when including the other variables total team size, effective team size, diversity of experience, and number of lead members. This was shown using a linear regression model.

Here we further underscore these results. In Fig. S5A we find that top teams have significantly higher experience than average teams, but this distinction disappears for $M \geq 7$. Figure S5B shows how E and S relate directly, independent of other quantities. We do see a significant increase in S as E grows, but the change in S is far smaller in magnitude than that of diversity D or number of leads L (shown in the main text). Moreover this effect is confounded with team size, and larger teams may even show a decrease in success as experience grows, although there are insufficient statistics to conclude a trend exists.

S6 Linear model with secondary team contributions

In Table S1 we present the linear model corresponding to that of the main text but augmented with two additional dependent variables: M_{ext}, the number of users who have submitted at least one pull request to the team, and W_{ext}, the number of submitted pull requests. These variables are both significantly correlated with team success S. Including them in the linear regression model did not change the significances of any other variables although it did alter the regression coefficients.

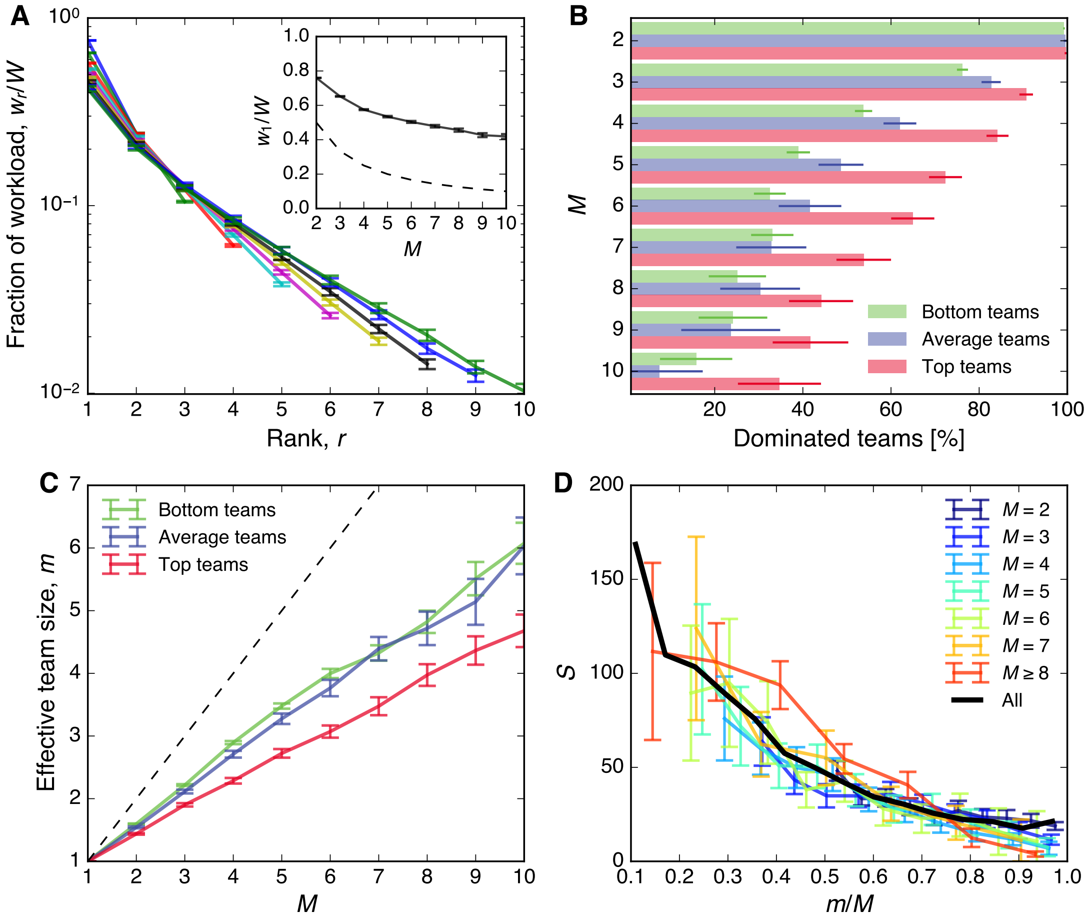

Figure S4: Teams are focused, and top teams are more focused than average teams of the same size.As per main text Fig. 2 but calculations now exclude outliers in S (above the 99th percentile). Our results do not change after removing outliers.

Variable x	Coefficient β_x		p
constant α	2.416×10^{-15}	$\pm$ 0.004489	1
Team size, M	-0.1165	$\pm$ 0.012856	$< 10^{-69}$
Eff. team size, m	0.0372	$\pm$ 0.011107	$< 10^{-10}$
Total work, W	0.0176	$\pm$ 0.004563	$< 10^{-13}$
Experience, E	-0.0032	$\pm$ 0.004520	0.16267
Diversity, D	0.0072	$\pm$ 0.005776	0.01506
Num. of leads, L	0.0534	$\pm$ 0.006371	$< 10^{-59}$
Age, T	0.0916	$\pm$ 0.004563	0
Num. pull requesters, M_{ext}	0.4684	$\pm$ 0.005480	0
Num. pull requests, W_{ext}	-0.0515	$\pm$ 0.005354	$< 10^{-78}$

Table S1: OLS regression model on team success, $S = \alpha + \beta_M M + \beta_m m + \beta_W W + \beta_E E + \beta_D D + \beta_L L + \beta_T T + \beta_{M_{\text{ext}}} M_{\text{ext}} + \beta_{W_{\text{ext}}} W_{\text{ext}}$. Outliers (above the 99th percentile in S) were filtered out to ensure they do not skew the model.

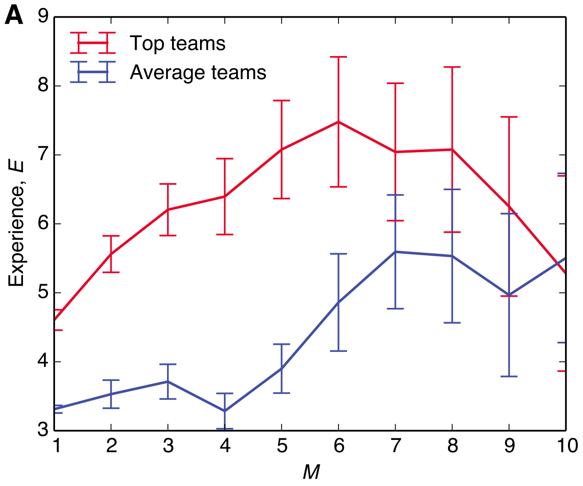

Figure S5: Experience weakly correlates with success. (**A**) Top teams have higher experience than average teams, but only for team sizes $M < 7$. (**B**) As experience grows, success grows on average, but the change in S is quite weak, compared to that of diversity and number of lead members (main text). Linear models also indicated that the effects of E are entirely due to the other quantities.
